# Single cell analysis of cancer cells using an improved RT-MLPA method has potential for cancer diagnosis and monitoring

**DOI:** 10.1038/srep16519

**Published:** 2015-11-12

**Authors:** L. Kvastad, B. Werne Solnestam, E. Johansson, A. O. Nygren, N. Laddach, P. Sahlén, S. Vickovic, Schirmer C. Bendigtsen, M. Aaserud, L. Floer, E. Borgen, C. Schwind, R. Himmelreich, D. Latta, J. Lundeberg

**Affiliations:** 1 Science for Life Laboratory, School of Biotechnology, Royal Institute of Technology (KTH), SE-171 65, Solna, Sweden; 2 MRC-Holland, Amsterdam, The Netherlands; 3 Agena Bioscience, San Diego, California; 4 Pathology Dept, Radiumhospitalet, Oslo University Hospital, Oslo, Norway; 5 Fraunhofer ICT-IMM, Mainz, Germany

## Abstract

Single cell analysis techniques have great potential in the cancer genomics field. The detection and characterization of circulating tumour cells are important for identifying metastatic disease at an early stage and monitoring it. This protocol is based on transcript profiling using Reverse Transcriptase Multiplex Ligation-dependent Probe Amplification (RT-MLPA), which is a specific method for simultaneous detection of multiple mRNA transcripts. Because of the small amount of (circulating) tumour cells, a pre-amplification reaction is performed after reverse transcription to generate a sufficient number of target molecules for the MLPA reaction. We designed a highly sensitive method for detecting and quantifying a panel of seven genes whose expression patterns are associated with breast cancer, and optimized the method for single cell analysis. For detection we used a fluorescence-dependent semi-quantitative method involving hybridization of unique barcodes to an array. We evaluated the method using three human breast cancer cell lines and identified specific gene expression profiles for each line. Furthermore, we applied the method to single cells and confirmed the heterogeneity of a cell population. Successful gene detection from cancer cells in human blood from metastatic breast cancer patients supports the use of RT-MLPA as a diagnostic tool for cancer genomics.

More than ten years ago Cristofanilli *et al.* used the CellSearch platform to show that circulating tumour cells (CTCs) have prognostic value in metastatic breast cancer patients^1^. Since then many methods to isolate, enumerate and analyse CTCs have been tested, with varying success. CTCs are defined as cancer cells that have detached from the primary tumour site and entered the peripheral blood circulation. The main challenge with CTCs is their low abundance, with as few as one single CTC per 10^6^–10^7^ leukocytes^2^. Isolation and enumeration of CTCs may be highly important not only for detecting metastatic disease early but also to monitor disease. Numerous efforts have been made in recent years to develop sensitive methods for detecting and quantifying CTCs, including use of microfluidic devices such as “CTC-chips”^3^,^4^,^5^,^6^,^7^ and immunomagnetic methods such as CellSearch^1^ and AdnaTest^8^. In addition to tumor cell enumeration of the samples, molecular characterization of the CTCs is believed to become of utmost clinical importance. Although reverse transcription quantitative PCR (RT-qPCR) is currently the main method used for molecular analysis of CTCs^9^, transcriptome analysis using RNA sequencing (RNA-seq) is advancing^10^.

Single-cell transcriptome profiling using RNA-seq enables analysis of gene expression in single cells in a mixed cell population. The method of single-cell RNA-seq has been applied to the analysis of CTCs of pancreatic^11^ and melanoma^10^ origin and although the technology has matured during the last couple of years, the method remains challenging. One observation is that gene expression may be high in one cell but low or even absent in another cell from the same population. A stochastic molecular process called “transcriptional burst”, in which the gene randomly switches back and forth between transcriptionally active and inactive states, may explain this variability^12^.

Genetic profiling using Reverse Transcription Polymerase Chain Reaction (RT-PCR) amplification of a limited set of genetic markers may offer a quick and affordable tool for analysing CTCs and improving diagnostic sensitivity. Several groups have designed and tested different multiplex PCR assays on cancer cells^13^,^14^,^15^,^16^,^17^. In this study, we have used a variant of the Reverse Transcriptase Multiplex Ligation-dependent Probe Amplification (RT-MLPA) assay developed at MRC-Holland. RT-MLPA^18^ is a variation on MLPA^19^ developed especially for mRNA profiling. Because of the small amount of tumour cells in this study, a pre-amplification reaction is performed after reverse transcription to generate a sufficient number of target molecules for the MLPA reaction. The MLPA method is based on sequence-specific probe hybridization to reverse transcribed RNA targets. Each MLPA probe consists of two or three target-specific oligonucleotides that are ligated after hybridization. A universal primer pair is used to amplify all ligated probes by PCR. As one MLPA probe oligonucleotide contains a specific barcode sequence, the amplification products can be distinguished by a fluorescence-dependent semi-quantitative detection method with hybridization of unique barcodes to an array.

In this study, we have chosen a panel of seven genes relevant to the molecular characterization of breast cancer cells. We have designed a set of MLPA probes for detecting and quantifying the gene expression, with single cell sensitivity. In the future we hope that our method will be useful for the molecular characterization of CTCs in patient blood samples.

## Results

### Multiplex analysis using an improved RT-MLPA protocol

**Figure 1:**
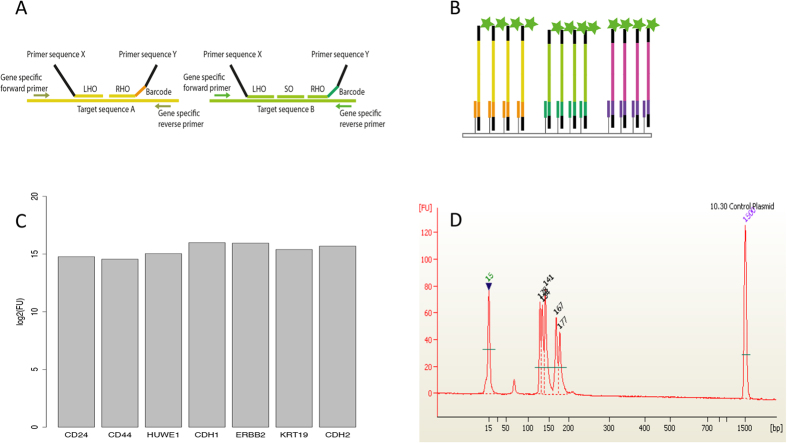
RT-MLPA probe layout and expression profiling. (**A**) Outline of the RT-MLPA probe design. The RT-MLPA probe mix contained either two or three oligonucleotides: a left hybridization oligonucleotide (LHO) consisting of a target-specific sequence and a universal primer sequence Y, a right hybridization oligonucleotide (RHO) consisting of a target-specific sequence, a unique barcode and a universal primer sequence X. Some of the probes also had a sequence-specific spanning oligonucleotide (SO) to increase specificity. Gene-specific forward and reverse primers were designed for each target gene. (**B**) Schematic layout of the array-based fluorescence-mediated detection using unique barcodes. The fluorescence molecules were visualized as green stars. (**C**) Expression profile of the control plasmid containing all seven targets. The data are expressed as median signal from fluorescence units (FU) detection normalized to *HUWE1* signals. (**D**) Expression profile of the control plasmid using capillary electrophoresis.

A sensitive, reproducible and sequence-specific method is valuable for detecting multiple targets in a single reaction. Our improved RT-MLPA protocol fullfils these criteria. The protocol starts with lysis of whole cells followed by reverse transcription. In order to enable sensitive detection of RNA transcripts, the original RT-MLPA assay was adjusted by introducing a pre-amplification step directly after the reverse transcription reaction. The pre-amplification reaction uses gene-specific primer pairs and amplifies specific targets during an optimized number of cycles of amplification. The subsequent MLPA reaction uses target-specific MLPA probes that consist of two synthetic oligonucleotides: a left hybridization oligonucleotide (LHO) and a right hybridization oligonucleotide (RHO). In some cases a third specific spanning oligonucleotide (SO) is used to ensure specificity in case of great homology to other regions. LHOs and RHOs each have gene-specific sequences together spanning exon-exon junctions to minimize amplification of genomic targets, and X and Y primer sequences, respectively. In this study, the RHO contains a unique barcode sequence used for detection. (See Fig. 1a and Supplementary Figure 1). The oligonucleotides are designed to anneal to adjacent sites on a target cDNA sequence and are ligated into a single DNA probe with the X and Y primer sites present at each end. Ligation will only occur if the target sequence is the perfect reverse complement of the probe sequence. The universal primer pair (X and Y) exponentially amplifies all ligated sequences in a single reaction using conventional PCR. The use of a universal primer pair preserves the relative number of fragments present before and after amplification. However, if the specific gene targets are not present in the cell, no hybridization and ligation occurs and there is no amplified product.

In this study, we have designed a RT-MLPA assay for simultaneous detection of seven genes, reported to have specific expression patterns during phases of breast cancer progression: *CD24*, *CD44*, *CDH2*, *CDH1*, *ERBB2*, *KRT19* and *HUWE1*. For solid tumours, the *CD44*^+^ phenotype is correlated with breast cancer initiator/stem cells^20^, while *CD24*^+^ cells are associated with poor survival of breast cancer patients^21^. *CDH1* (also known as epithelial cadherin, or E-cadherin) and *CDH2* (also called neural cadherin, or N-cadherin) have important functions in cell-cell adhesion. A switch in expression from *CDH1* to *CDH2* contributes to increased tumour cell migration, invasion and metastasis and is reported as part of the process called epithelial mesenchymal transistion (EMT)^22^,^23^,^24^. *ERBB2* (also known as *HER2* - human epidermal growth factor receptor 2) plays an important role in human malignancies and is overexpressed in approximately 10–40% of human breast cancers^25^,^26^. Cytokeratin 19 (KRT19) mRNA is the most popular marker for CTC detection in breast cancer patients by RT-PCR methods^27^,^28^. We included *HUWE1* as a reference gene, since it is expressed relatively strongly in many tissues^29^. We designed specific primers and oligonucleotides for each gene. The resulting amplicons each have unique barcodes and varying lengths (115–170 bp) to enable each product to be distinguished. To detect amplicons by size, standard chip-based capillary electrophoresis was used, while barcode-based detection involved specific binding to an array followed by fluorescence visualization (see Fig. 1b). For the latter we performed MLPA amplification using the universal primer pair modified with Cy3-fluorophore and biotin (see Supplementary Table 1 for probe, primer and barcode sequences). Capillary electrophoresis only gives an estimate of the target genes’ expression while array-based detection using barcodes gives a highly accurate detection signal for each target. To test the efficiency of the MLPA probes, a control plasmid mixture containing all seven target genes was analysed and found to give a signal from all gene targets (Fig. 1c,d). Each gene target was also tested individually and analysed using capillary electrophoresis (Supplementary Figure 2) and array-based fluorescence detection (data not shown). Furthermore, a correction factor was calculated using synthetic templates for each target to correct for differences in probe efficiency (see Methods for more details).

### Optimization of the RT-MLPA protocol towards single cell sensitivity

**Figure 2:**
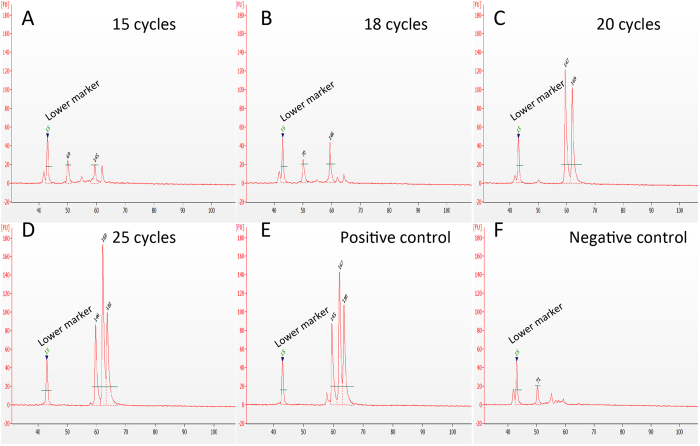
Length distribution of RT-MLPA products from pre-amplification optimization. Expression profiles visualized by gel electropherograms generated by Bioanalyzer obtained from: (**A**–**D**) 100 pg total RNA from MCF-7 cells amplified with 15, 18, 20 and 25 cycles in the pre-amplification step. (**E**) Positive control (1 ng total RNA from MCF-7 cells) amplified with 25 cycles. (**F**) Negative control (water).

When analysing small amounts of RNA, as for single cells, it is important to have a method that can sensitively and accurately discriminate the signal of a single cell from its background. Here we evaluated both the optimal number of pre-amplification cycles using gene-specific primers and the optimal number of cycles in the MLPA amplification using universal primers. First, we estimated the total amount of RNA in a human breast cancer cell (SKBR-3) to accurately dilute total RNA to single-cell level. The results show that a cell contains 30–40 pg of total RNA (Supplementary Table 2), which is consistent with previous estimates of the total amount of RNA in a small eukaryotic cell^10^. We addressed the sensitivity of the pre-amplification method by evaluating the optimal number of cycles in the pre-amplification reaction using quantitative PCR (qPCR) and total RNA from the human breast cancer cell line MCF-7 diluted to near single-cell level (100 pg). Based on the median of the mean quantification cycle (Cq) from a dilution series (data not shown), the number of pre-amplification cycles was increased from 15 to 25 and, the yield was compared with a positive control of 1 ng total RNA (Fig. 2). The results showed that the optimal number of pre-amplification cycles for single cell levels of RNA was 25 when using a fixed number of amplification cycles.

**Figure 3:**
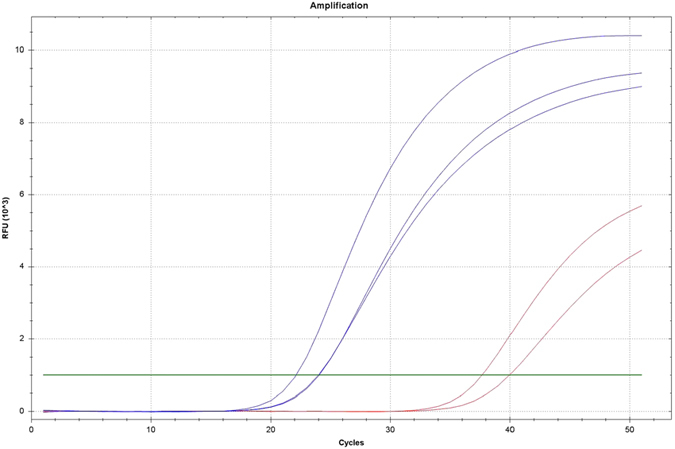
Quantitative PCR using single cells. Curves from quantitative PCR of three single MCF-7 cells (blue) and two negative controls (red). Green line indicate automatic threshold.

The next step towards single cell sensitivity was to evaluate the number of amplification cycles required for the MLPA step. Whole single MCF-7 cells were picked using laser capture micro-dissection (LCM) and analysed with qPCR (Fig. 3). The results showed a clear separation between single cells and the negative control and a total number of 35 cycles was deemed optimal and adopted in the protocol.

Variation in RNA quality is a frequent issue in transcript studies. The RNA integrity number (RIN) is a commonly used measure of the quality of extracted RNA, ranging from RIN 1 (completely degraded) to RIN 10 (completely intact RNA). To assess the robustness of the RT-MLPA protocol, total RNA from MCF-7 cells of different quality were tested and analysed using chip-based capillary electrophoresis. A clear pattern of four peaks was detected between 115–170 bp for all samples with RIN values ranging from 1.30 to 9.80 (Supplementary Figure 3).

### Evaluating the gene panel and comparing RT-MLPA results with RNA sequencing data

**Figure 4:**
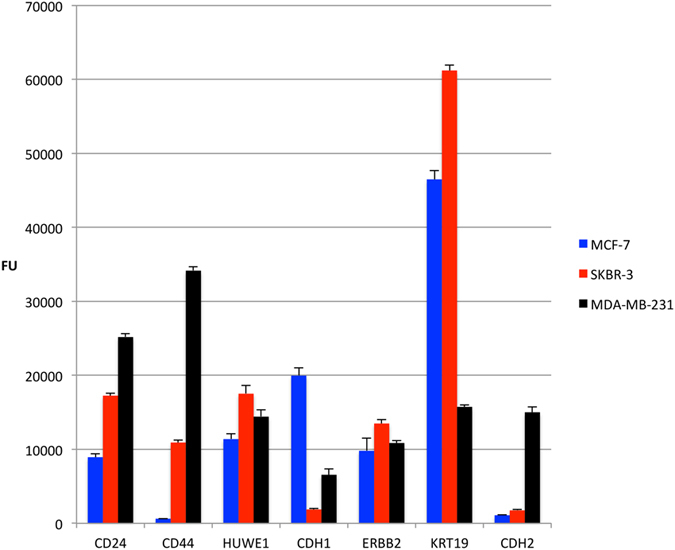
RT-MLPA of three human breast cancer cell lines. Expression profiles of three breast cancer cell lines MCF-7 (blue), SKBR-3 (red) and MDA-MB-231 (black) for the seven genes of the breast cancer panel. RT-MLPA results were measured in fluorescence units and bars represent standard deviation.

For evaluation of the gene panel, total RNA extracts from three human breast cancer cell lines (MCF-7, SKBR-3 and MDA-MB-231) were used in the optimized RT-MLPA protocol. The results were analysed using the array-based fluorescence detection method and showed a specific gene expression profile for each cell line (Fig. 4), corresponding capillary electrophoresis data (Supplementary Figure 4).

**Figure 5:**
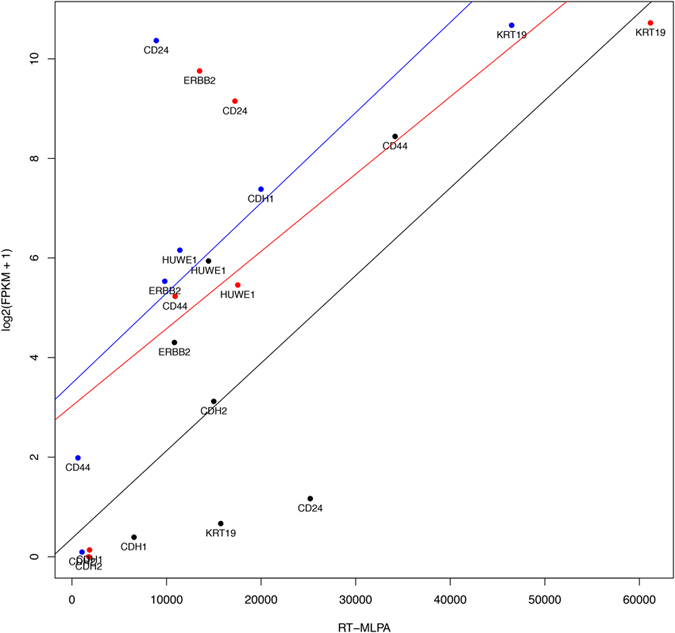
Scatterplot of all seven genes from the three cell lines. Scatterplot of the gene expression estimates for three cell lines; SKBR-3 (red), MCF-7 (blue) and MDA-MB-231 (black)—obtained using RNA-seq and RT-MLPA. Lines show the linear regression curve for each cell line.

The RT-MLPA result for each gene in the panel was compared to the normalized gene expression value (FPKM—fragments per kilobase of exon per million fragments mapped). The RNA-seq data were correlated to the RT-MLPA results for each cell line and displayed in a scatterplot (Fig. 5). The Pearson correlation between the expression levels was high, at 0.71, 0.89 and 0.77 for the MCF-7, SKBR-3 and MDA-MB-231 cell lines, respectively.

### Single-cell sensitivity

**Figure 6:**
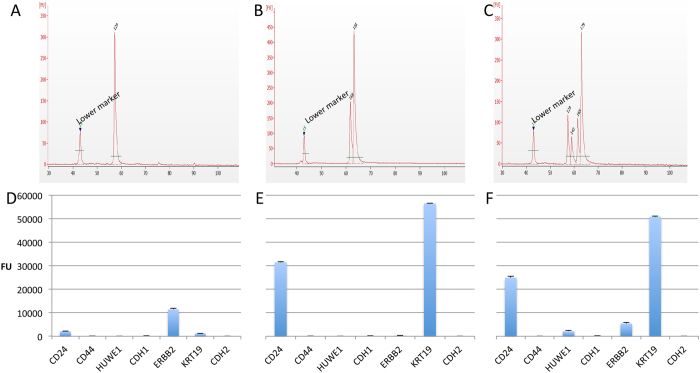
Single-cell level RT-MLPA. Results of analysis of three single SKBR-3 cells bars represent standard deviation: (**A**–**C**) Gel electropherograms from Bioanalyzer and (**D**–**F**) results of corresponding array-based fluorescence detection.

In order to test the sensitivity of the RT-MLPA method, single cells from a breast cancer cell line (SKBR-3) were collected by LCM and used in the RT-MLPA assay. The resulting products were analysed using capillary electrophoresis and by the array-based fluorescence method. Typically, a different gene expression pattern was observed for each single cell analysed. The results from three single SKBR-3 cells are shown in Fig. 6.

By combining RT-MLPA results from nine single SKBR-3 cells and comparing the average with the results from bulk sample of SKBR-3 (100 ng), we saw comparable signals from the three most highly expressed genes (*CD24*, *ERBB2* and *KRT19*). Genes expressed at lower levels were not detected in the averaged single cell data but were detected in bulk sample (Supplementary Figure 5).

### RT-MLPA on tumour cells in human blood samples

A method for CTC detection requires single cell sensitivity and needs to also distinguish the cancer signal above background. A common method for enriching tumour cells in blood samples is the use of immunomagnetic separation (IMS) with anti-EpCAM (epithelial cell adhesion molecule) antibody-coated magnetic beads^30^. First, we investigated whether anti-EpCAM coated beads adhere preferentially to MCF-7 cells in the presence of mononuclear cells (MNCs) and red blood cells. Samples included both erythrocytes (red blood cells) and MNCs spiked with MCF-7 cells. The anti-EpCAM coated beads were observed to adhere to MCF-7 cells (Supplementary Figure 6).

**Figure 7:**
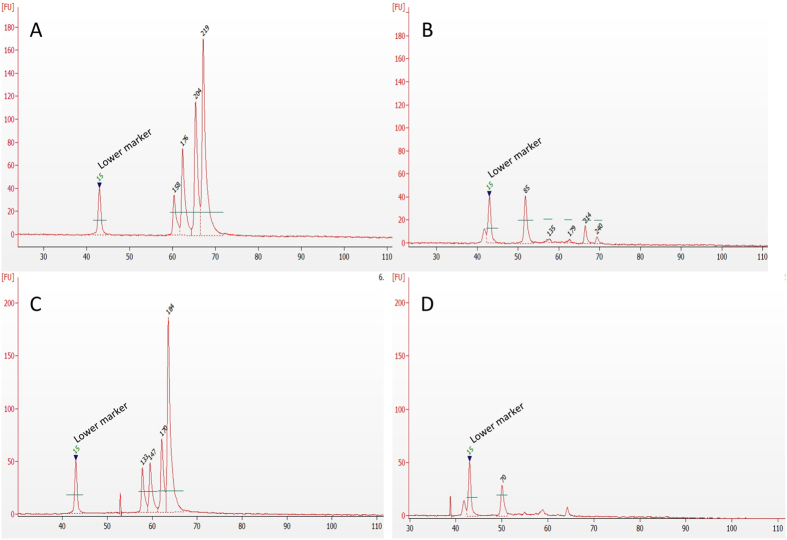
Length distribution of RT-MLPA products from human blood samples. Gel electropherograms from Bioanalyzer with and without spiked cancer cells. (**A**) Blood from healthy donor spiked with 300 000 MCF-7 cells. (**B**) Blood from healthy donor. (**C**) Formaldehyde-fixed blood from healthy donor spiked with 300 000 MCF-7 cells. (**D**) Formaldehyde-fixed blood from healthy donor.

Secondly, we evaluated the compatibility of the RT-MLPA protocol with human samples and the specificity of the assay, by analysing blood from healthy donors and blood from healthy donors spiked with cancer cells (MCF-7). All samples were positively enriched by IMS using anti-EpCAM antibody-coated magnetic beads before the RT-MLPA. Following the RT-MLPA reaction products were analysed using capillary electrophoresis (Fig. 7a,b) and the array-based fluorescence detection method (data not shown). Correct gene signals were only observed in the spiked blood samples.

In a clinical setting there may be a time lag between sample collection and analysis. It is therefore important to consider fixation and/or snap freezing of samples. To investigate the potential influence of formaldehyde fixation on the results of RT-MLPA, we analysed human blood samples spiked with MCF-7 cells followed by fixation using 2% formaldehyde in PBS (pH 7.4). As previously, the samples were treated with IMS and anti-EPCAM antibody-coated beads before RT-MLPA and analysed using capillary electrophoresis (Fig. 7c,d). Similarly to the non-fixed samples, target gene-specific signals were only observed in the samples containing cancer cells.

### RT-MLPA on circulating tumour cells in patient samples

**Figure 8:**
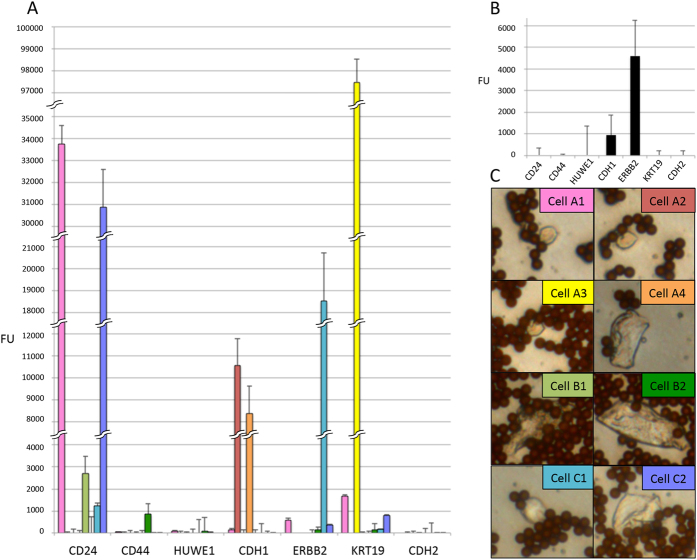
RT-MLPA on cells from metastatic breast cancer patients. Results of analysis from four metastatic breast cancer patients, bars represent standard deviation: (**A**) Expression patterns of single cells from three patients. Single cells are colour coded. (**B**) Expression pattern from one patient, IMS done using AdnaSelect beads, cells analysed in bulk. (**C**) Images of picked single cells from three patients, IMS done using AdnaSelect beads, image at 40× magnification. Single cells are colour and alphabetically coded to individual cells and patients respectively.

Blood samples from metastatic breast cancer patients were screened using the CellSearch system to confirm the presence of CTCs in blood and the following numbers of CTCs were detected in the corresponding patients using 7.5 ml blood; patient A (12), patient B (12), patient C (9) and patient D (62). We validated the RT-MLPA protocol on these patients using 7.5 ml of blood (patient A–C) and 4 ml of blood (patient D). Single cells isolated by IMS and LCM using patients A to C and by using the entire sample (bulk) from patient D (Fig. 8). Direct staining with AdnaSelect anti-EpCAM and anti-MUC1 antibody-coated beads was used to enable detection of CTCs during LCM. Cells with attached beads were considered to be possible CTCs. In patients A-C, between 2 and 4 single cells showed RT-MLPA signal above noise level (Fig. 8a,c), and in patient D two genes were detected when analysing the entire IMS treated sample (Fig. 8b).

## Discussion

We have designed a highly sensitive and robust RT-MLPA assay for easy and reliable expression profiling of a selected gene panel for breast cancer and optimized the protocol for use on single cells. The selected panel allows quick and efficient characterization of multiple gene expression per reaction on a single-cell level.

Optimization of the RT-MLPA protocol for single cell sensitivity showed that 25 pre-amplification cycles were needed for low amounts of total RNA to gain a signal comparable to the positive control, when using chip-based capillary electrophoresis or fluorescence-dependent semi-quantitative method with hybridization of unique barcodes. Furthermore, validation of the number of cycles in the final amplification of the RT-MLPA showed that 35 cycles generated a detectable signal above background, equal to the number of cycles in the standard MLPA protocol. The high total number of amplification cycles required indicated the relatively low efficiency of the ligation of the MLPA oligonucleotides, and that multiple ligation events, as in the case of the probes with a spanning oligonucleotide, result in poorer ligation. However, this could also be due to the hybridization time and/or the amount of oligonucleotides in the reaction mix. The differences in probe efficiency were adjusted for by using a calculated correlation factor (Supplementary Table 3). The improved protocol was evaluated on total RNA from three human breast cancer cell lines (MCF-7, SKBR-3 and MDA-MB-231) and showed a specific gene expression profile for each cell line. For example, expression of the gene *CDH1* was very high in MCF-7 cells, but low or absent in SKBR-3 and MDA-MB-231 cells. Similarly the expression of *KRT19* was high in MCF-7 and SKBR-3 cells, but low in MDA-MB-231 cells.

These results were confirmed by whole transcriptome profiling of all three cell lines using RNA-seq. Comparison of the RT-MLPA results and the normalized gene expression value from the RNA-seq for each of the selected gene of the panel confirmed that the same genes were found to be highly expressed by both methods. The two data sets showed high correlation with coefficients between 0.71 and 0.89.

The single-cell sensitivity of the RT-MLPA assay is of utmost importance for the analysis of CTCs in patient blood samples. We have successfully applied the RT-MLPA assay to single cells. We typically observed gene expression patterns of one to four genes. Although it is possible that some transcripts may be lost due to sample handling and degradation, we think that the different peak patterns could be due to transcriptional ‘bursting’, in which transcriptional activity varies markedly between individual cells^12^ within a heterogeneous cell population. Cell heterogeneity is an important consideration in single cell analysis. However, comparing combined results from the single cell experiments with the positive control showed similar expression patterns for the three highly expressed genes in SKBR-3 (*CD24*, *ERBB2* and *KRT19*), whereas transcripts of genes expressed at lower levels were only detected in the positive control. These results show the RT-MLPA assay to be sensitive enough to detect different gene expression patterns in single cells.

To assess its usefulness as a diagnostic tool we evaluated the RT-MLPA assay’s utility for analysing CTCs enriched from patient blood samples. We fixed the samples with formaldehyde prior to analysis in order to prevent RNA degradation that could occur during the time lag between sampling at the clinic and analysis^31^. Results from human blood cells spiked with MCF-7 cells support the use of RT-MLPA as a convenient diagnostic tool and indicate that patient samples can be fixed prior to analysis without compromising the results. The analysis of samples of low and high quality RNA supports its use in a variety of clinical and laboratory settings where it would be expected to yield comparable results, and opens up the possibility of analysing formalin-fixed, paraffin-embedded samples of solid tumours.

Our study shows that the RT-MLPA protocol has potential for expression analysis of CTCs from metastatic breast cancer patients. In our single CTC experiments in the patient material we observed expression of both *KRT19* and *ERBB2*, which expression has previously been associated with metastatic breast cancer^27^,^28^. Analysis of single cells from patient samples showed variable results often with signal from only one gene in our panel. This observation of gene expression in single cells was also observed in our experiments using single cells from cancer cell lines. In contrast when multiple cells (cancer cell line or CTCs) were analysed we observed co-expression of multiple markers possibly indicating that biomarkers of individual single cell are merged into a consensus gene expression profile.

In conclusion, our RT-MLPA assay makes a valuable contribution to expression profiling of breast cancer cells at the single-cell level. The combination of RT-MLPA as a diagnostic tool, with an enrichment method for CTCs, could in the future offer cancer patients and physicians the potential for quick and affordable monitoring of the disease progression and potential relapse.

## Methods

### Design of probes, primers and barcodes

All probes and primers were designed and quality tested by MRC-Holland according to their standardized protocols. The 5′ modified forward and reverse primers were ordered from MWG. Unique barcodes for each gene target were designed and tested *in silico* and ordered from MWG (see Supplementary Table 1 for sequences).

### Cell cultivation

Three human breast cancer cell lines; MCF-7 (Dr. Emma Lundberg, Royal Institute of Technology), SKBR-3 (Cell-Lines Service, Eppelheim, Germany) and MDA-MB-231 (Cell-Lines Service, Eppelheim, Germany)—were cultivated at 37 °C in a 5% CO_2_ environment in media suggested by the provider. Cells were harvested during log-phase growth (70–90% confluency).

### RNA extraction

Total RNA was extracted and prepared using the RNeasy Mini extraction kit (Qiagen, Hilden, Germany) according to the manufacturer’s instructions. Quality of the total RNA was determined using RNA 6000 Nano chip on the 2100 Bioanalyzer automated electrophoresis system (Agilent Technologies Inc., Santa Clara, CA, USA) or Experion automated electrophoresis system (Bio-Rad Laboratories, Hercules, CA, USA) with standard sensitivity RNA chip. Concentration of the total RNA was determined using the Qubit RNA BR assay (Life Technologies, Carlsbad, CA, USA).

### RT-MLPA single cell assay—reverse transcription and pre-amplification

The RT-MLPA assay is outlined in Supplementary Figure 1. The total RNA amount used in the experiments was 100 ng unless otherwise stated. For dilution and pipetting of RNA and amplified cDNA special low binding tips (Biotix, San Diego, CA, USA) and DNA LoBind tubes (Eppendorf, Hamburg, Germany) were used. All PCR reactions were carried out at a total volume of 20 μl unless otherwise stated. The protocol was performed as follows: RNA samples were denatured for 1 min (80 °C), and cells were dissolved in lysis buffer (0.2% Tween-20, 0.2% NP-40, 19 mM Tris-HCl pH 7.5 and 1 ng/μl single stranded viral vector M13mp18), heated for 2 min (80 °C) and placed on ice. Reagents for reverse transcription and pre-amplification were added yielding a final concentration of 50 mM Tris-HCl (pH 8.3), 75 mM KCl, 3 mM MgCl_2_, 10 mM DTT, 4⋅0.1 mM dNTPs, 5% DMSO, 8 μg BSA, 40 units Reverse Transcriptase (Promega, Madison, WI, USA), 0.4 units of SALSA polymerase (MRC-Holland, Amsterdam, The Netherlands) and 0.048 units of Pfu DNA Polymerase (Promega, Madison, WI, USA). For primer sequences and concentrations see Supplementary Table 1. Reverse transcription was performed for 10 min (42 °C), followed by 25 cycles of PCR (30 s 95 °C, 5 min 54 °C, and 60 s 72 °C).

### RT-MLPA single cell assay—multiplex ligation-dependent probe amplification

From the pre-amplification mixture 2.5 μl was used for MLPA. An initial incubation of 5 min (98 °C) to inactivate the polymerase was followed by addition of 0.75 μl MLPA Buffer and 0.75 μl of 3 fmol of each target-specific oligonucleotide (both from MRC-Holland, Amsterdam, The Netherlands). The resulting mixture was incubated at 1 min (95 °C) to denature the probes, followed by hybridization for 60 min (60 °C). Combined ligation and multiplex amplification was performed by adding 16 μl of a mixture containing 1.5 μl each of Ligase Buffer A and B, 1 μl Ligase-65, 1.2 units SALSA Polymerase (all from MRC-Holland, Amsterdam, the Netherlands), 4 nmol dNTPs, 5 pmol each of Cy3-labelled primer Y and biotinylated primer X, and 4 μl Q-solution (Qiagen, Hilden, Germany). Ligation was performed for 4 minutes (54 °C) followed by 35 cycles of PCR (30 seconds at 95 °C, 30 seconds at 60 °C, and 60 seconds at 72 °C) and 10 minutes (72 °C).

### Detection of gene specific amplicons based on length and barcodes

Amplified MLPA fragments were analysed and quantified using two different methods. The first was based on length and used DNA 1000 chip on the 2100 Bioanalyzer automated electrophoresis system (Agilent Technologies Inc., Santa Clara, CA, USA). The second method was a fluorescence-dependent semi-quantitative method for hybridization of unique barcodes to an array (Supplementary Figure 7). The array consists of one row of spots for each MLPA probe, printed with capture probes complementary to the barcodes of the MLPA probes, with a total of 5 to 8 spots per row. Please see supplementary methods for more information.

### Single cell isolation

Whole cells were detached from the growth surface and re-suspended in PBS buffer. Single cells were isolated using the MMI CellCut tool for LCM from MMI (Molecular Machines and Industries, Zürich, Switzerland) coupled to an IX81F-3 microscope with an IX2-UCB external power supply (Olympus, Shinjuku, Japan). Samples prepared for transportation was smeared onto a LCM slide, dried at 60 °C for 45 minutes and stored at −80 °C.

### Tumour cell spiking in blood and mononuclear cell fractions

The specificity of the RT-MLPA technique was analysed using tumour cells in a background of blood cells (MNCs, whole blood or whole blood diluted 1:1 in PBS and spiked with MCF-7 cells). Blood samples from healthy donors were drawn in EDTA tubes (Beckton Dickinson and Company, Franklin Lakes, NJ, USA), transported and stored at room temperature, and processed within 12 hours. MNCs were isolated from 7.5 ml whole blood diluted 1:1 in PBS using Lymphoprep separation medium (Fresenius Kabi, Bad Homburg, Germany), density 1.077 g/ml, and 50 ml Leucosep tubes (Greiner Bio-One, Frickenhausen, Germany) according to the manufacturer’s instructions. The MNC cells were washed twice with MCF-7 culture medium and centrifuged at low RCF. After the final wash, the MNCs were dissolved in 200 μl PBS and spiked with approximately 1 million MCF-7 cells and centrifuged. The supernatant was discarded and cells were dissolved in 50 μl PBS. In addition, whole blood samples (500 μl whole blood and 500 μl whole blood diluted 1:1 in PBS) were spiked with 300 000 MCF-7 cells. Tumour cells were isolated using magnetic beads coupled to an antibody targeting the epithelial marker EpCAM (CELLection Epithelial Enrich Dynabeads, Life Technologies, Carlsbad, CA, USA) according to the manufacturer’s instructions. Total RNA was extracted from the samples using the RNeasy FFPE mini kit (Qiagen, Hilden, Germany). Before RNA extraction the samples were spun down and the supernatant pipetted off. The deparaffination steps were excluded from the protocol. RT-MLPA was then performed on the extracted RNA.

### Experimental setup for patient samples

The impact of sample preparation and transportation was tested using MCF-7 and SKBR-3 cancer cells, cultivation and single cell isolation was done as previously described in this article. Negative selection was done for only the spiking experiment with SKBR-3 cancer cells using RosetteSep CTC enrichment cocktail containing anti-CD56 (StemCell Technologies), according to manufactures instruction. Immunomagnetic selection by AdnaSelect (BreastCancerSelect, QIAGEN Hannover) was done according to manufactures instruction, except for 150 μl beads were used for 7.5 ml blood.

In our experimental setup for cancer patients the samples were collected at one location and later shipped to a second location for analysis. To determine the impact of sample preparation and transportation compatible with RT-MLPA and clinical practice for these patients, single cancer cells (MCF-7) were analysed and pooled data from eight single cells yielded a high positive Pearson correlation of 0,77 compared to RNA-seq data (Supplementary Figure 8). To establish the impact of IMS before LCM, blood from healthy donor was spiked with cancer cells (SKBR-3) and IMS performed using AdnaSelect beads. We detected single cells having a SKBR-3 like expression profile, with Pearson correlation >0,85 compared to RNA-seq data (Supplementary Figure 9).

In total four metastatic breast carcinoma patients undergoing cancer therapy and/or clinical examinations at Oslo University Hospital were included. Written informed consent was obtained from all patients, and the Regional Ethical Committee approved the study. From each patient 30 ml peripheral blood was sampled and divided into 7.5 ml aliquots. One aliquot was used for analysis with the FDA-approved CellSearch system (Janssen Diagnostics LLC), enumeration of detected CTC was performed according to standard CellSearch CTC definition^32^. Another aliquot was subjected to immunomagnetic selection by AdnaSelect (BreastCancerSelect, QIAGEN Hannover) as previously described in this article, except for patient A-D were samples were washed using PBS with 0.1% BSA instead of Adna washing buffer. Also, for patient D 100 μl beads were used for 4 ml blood, incubation of beads in blood was done for 15 minutes and in washing buffer for 7.5 minutes. RT-MLPA was done as previously described in this article, except a different lysis buffer was used for patient D (0.002% Brij 58, 1 mM EDTA, 10 mM Tris-HCl pH 7.5, 0.5 mM Imidazol and 1 ng/μl single stranded viral vector M13mp18). Also, ligation and multiplex amplification was performed separately as follows. Ligation was performed by adding 8 μl of a mixture containing 1.5 μl each of Ligase Buffer A and B and 1 μl Ligase-65. Ligation reaction was performed at temperatures as follows; ligation 4 minutes (54 °C), enzyme inactivation 5 minutes (98 °C), hold (25 °C). The multiplex amplification was performed by adding 8 μl of a mixture containing 1.2 units SALSA Polymerase (all from MRC-Holland, Amsterdam, the Netherlands), 4 nmol dNTPs, 5 pmol each of Cy3-labelled primer Y and biotinylated primer X, and 4 μl Q-solution (Qiagen, Hilden, Germany). The multiplex amplification reaction was performed at temperatures as follows; 35 cycles of PCR (30 seconds at 95 °C, 30 seconds at 60 °C, and 60 seconds at 72 °C) and 10 minutes (72 °C). For patient D the CTCs were analysed in bulk, immunomagnetic beads were removed by magnet, RT-MLPA performed as previously described in this article, except during the multiplex amplification reaction primer X and Y were unlabelled. To label the sample the following was performed. The sample was diluted 30 times in water and reagents were added yielding a final concentration of 50 mM Tris-HCl (pH 8.3), 75 mM KCl, 3 mM MgCl_2_, 10 mM DTT, 1.2 units SALSA Polymerase (all from MRC-Holland, Amsterdam, the Netherlands), 4 nmol dNTPs, 5 pmol each of Cy3-labelled primer Y and biotinylated primer X, and 4 μl Q-solution (Qiagen, Hilden, Germany). Then the multiplex amplification reaction was performed at temperatures as follows; 35 cycles of PCR (30 seconds at 95 °C, 30 seconds at 60 °C, and 60 seconds at 72 °C) and 10 minutes (72 °C).

## Additional Information

**How to cite this article**: Kvastad, L. *et al.* Single cell analysis of cancer cells using an improved RT-MLPA method has potential for cancer diagnosis and monitoring. *Sci. Rep.* **5**, 16519; doi: 10.1038/srep16519 (2015).

## Supplementary Material

Supplementary Information
