# Supplementary material for: Single cell analysis of cancer cells using an improved RT-MLPA method has potential for cancer diagnosis and monitoring
Source: Sci Rep. 2015 Nov 12;5:16519. doi: 10.1038/srep16519 (PMC4642268; doi:10.1038/srep16519)
Supplement: Supplementary Information [file srep16519-s1.pdf]

Supplementary Information

Single cell analysis of cancer cells using an improved RT-MLPA method has potential for cancer diagnosis and monitoring

Kvastad L[1§], Werne Solnestam B[1§], Johansson E[1], Nygren A O[2,3], Laddach N[2], Sahlén P[1], Vickovic S[1], Bendigtsen Schirmer C[4], Aaserud M[4], Floer L[4], Borgen E[4], Schwind C[5], Himmelreich R[5], Latta D[5], Lundeberg J[1*]

[1]Science for Life Laboratory, School of Biotechnology, Royal Institute of Technology (KTH), SE-171 65, Solna, Sweden. [2]MRC-Holland, Amsterdam, The Netherlands. [3]Agena Bioscience, San Diego, California. [4]Pathology Dept, Radiumhospitalet, Oslo University Hospital, Oslo, Norway. [5]Fraunhofer ICT-IMM, Mainz, Germany.

[§]Authors contributed equally

[*]Corresponding author

Corresponding author:
Joakim Lundeberg
Science for Life Laboratory
Royal Institute of Technology (KTH)
School of Biotechnology
SE-171 65 Solna, Sweden
+46-8-524 814 69
joakim.lundeberg@scilifelab.se

Supplementary Figures

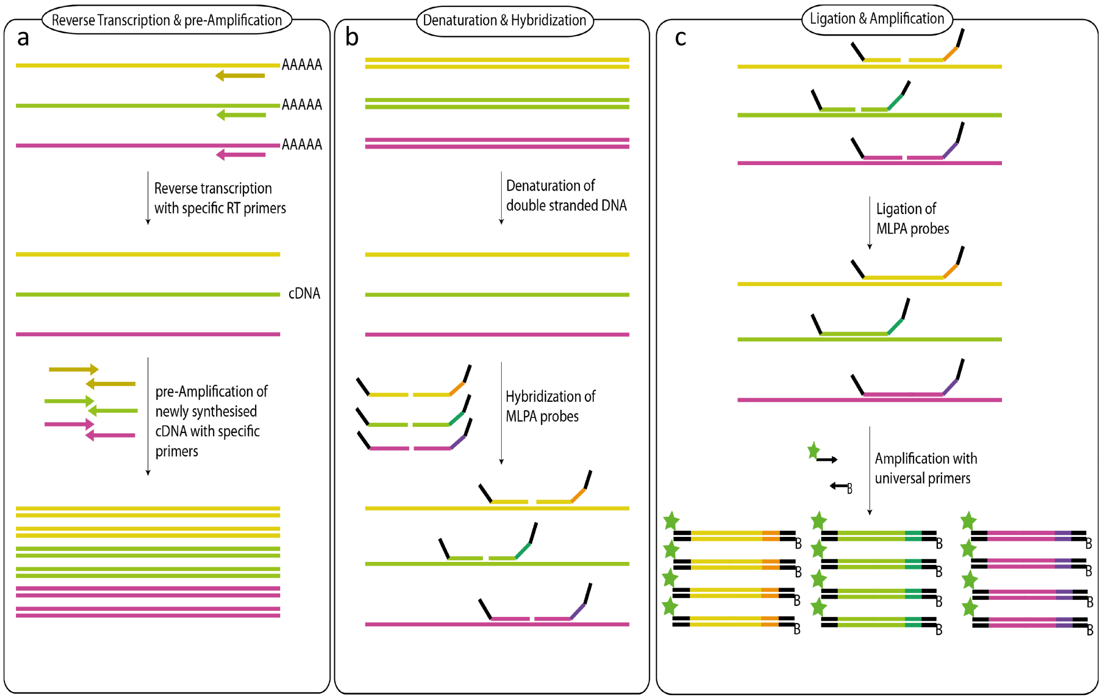

Supplementary Figure S1. Outline of RT-MLPA protocol

a) Reverse transcription and pre-amplification. b) Denaturation and hybridization of specific MLPA probes. c) Ligation and multiplex amplification.

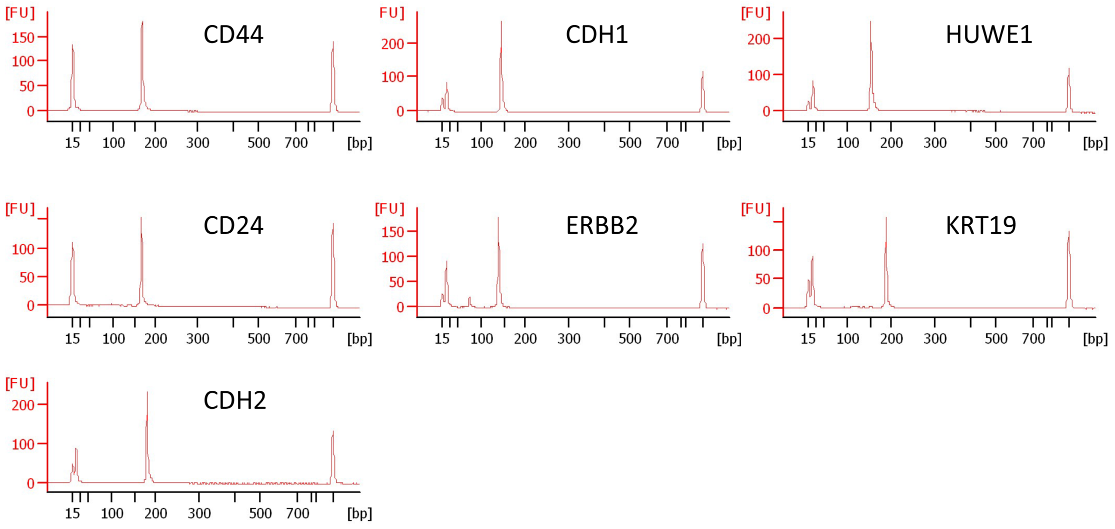

Supplementary Figure S2. Individual analysis of genes in panel detected by capillary electrophoresis

Each gene in the gene panel has been tested individually and shows the correct peak for each target.

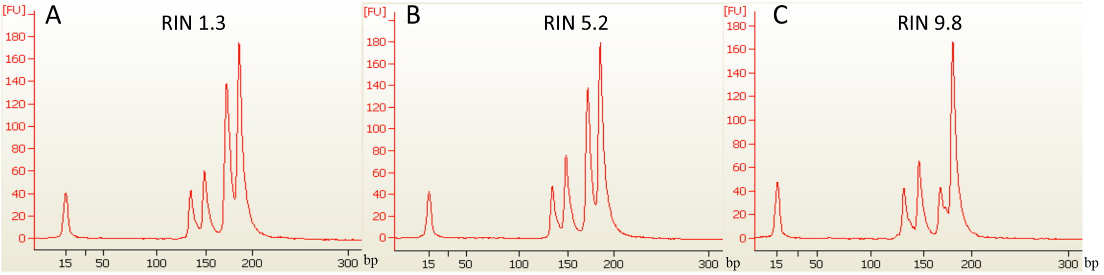

Supplementary Figure S3. Length distribution of RT-MLPA products from total RNA in different states of degradation.
a) RIN 1.3 b) RIN 5.2 c) RIN 9.8

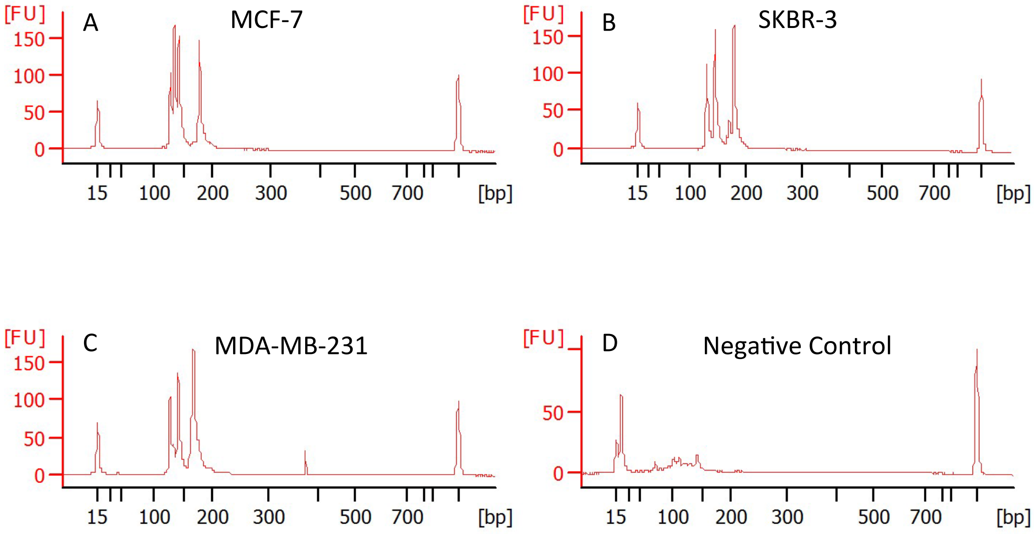

Supplementary Figure S4. Length distribution of RT-MLPA products from total RNA from three cell lines and negative control.
a) MCF-7 b) SKBR-3 c) MDA.MB.231 d) Negative control

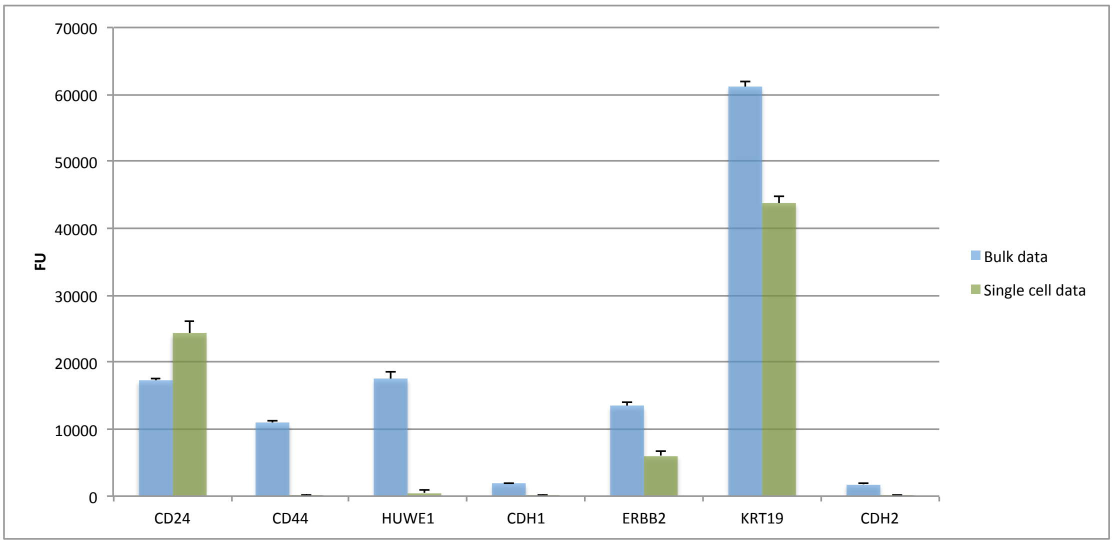

Supplementary Figure S5. Average signals from single cells compared with bulk sample

Graph showing average RT-MLPA results from nine single SKBR-3 cells (green) and a bulk SKBR-3 control (100 ng) (blue), bars represent standard deviation.

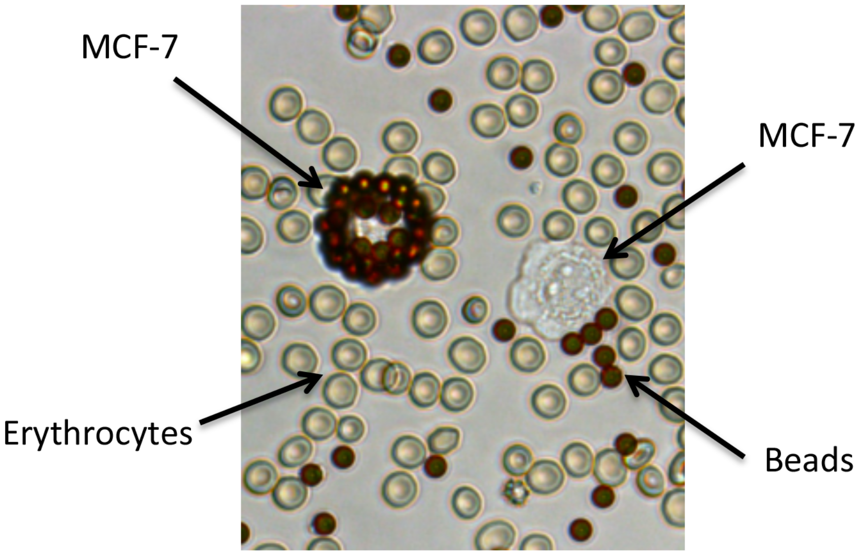

Supplementary Figure S6. Microscopy images of cells during immunomagnetic selection (IMS)

Samples during IMS using EpCAM positive beads, 40 x magnification. Blood spiked with MCF-7 cells. The tumour cell to the left has higher EpCAM expression and attracts more beads than the tumour cell to the right.

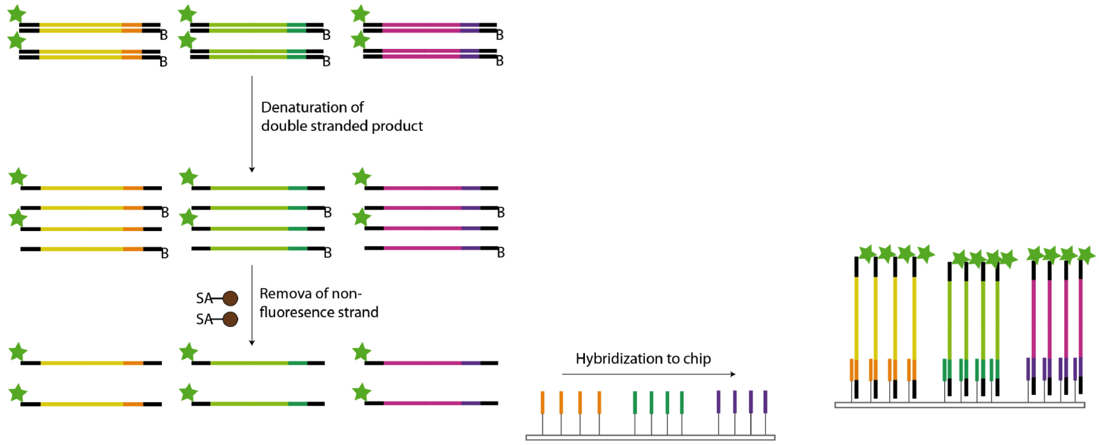

Supplementary Figure S7. Outline of fluorescence-dependent semi-quantitative detection method

Schematic outline of the fluorescence-dependent semi quantitative detection method used for specific identification of gene targets.

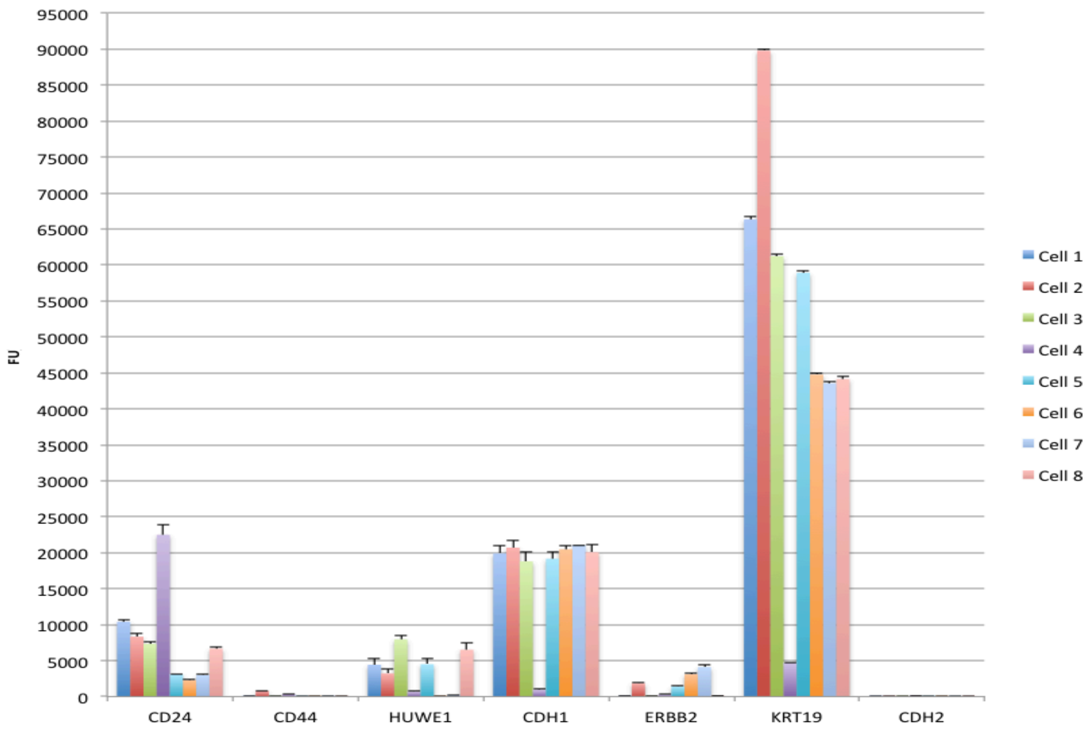

Supplementary Figure S8. Signals from single cells after transportation and LCM

Graph showing RT-MLPA results from eight single MCF-7 cells, bars represent standard deviation. Pooled data from all single cells yields a high positive Pearson correlation of 0,77 compared to RNA-seq data.

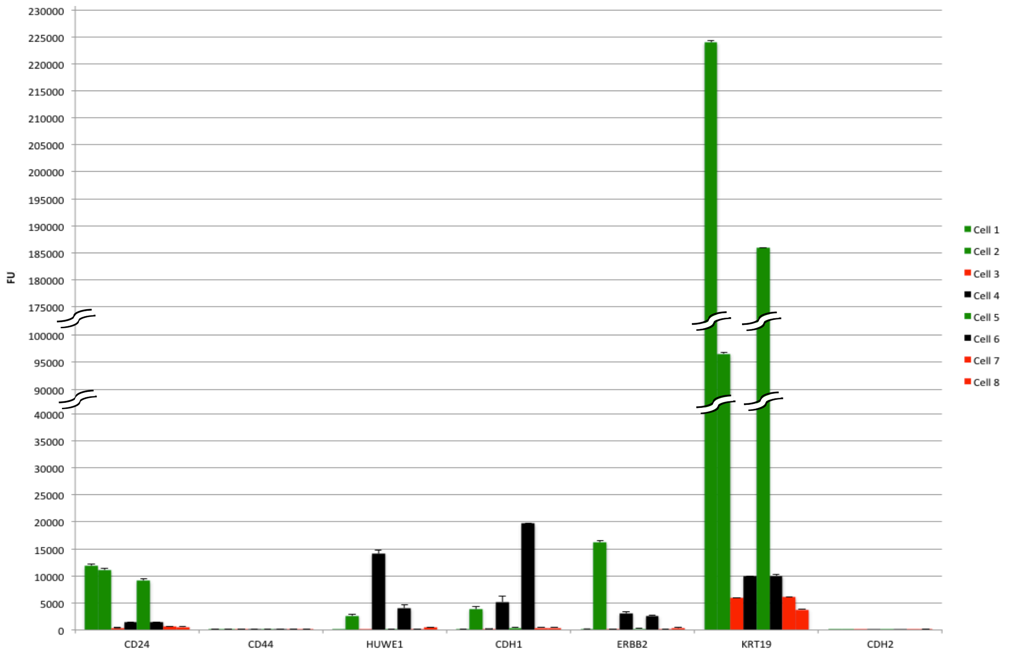

Supplementary Figure S9. Signals from single cells after IMS, transportation and LCM

Graph showing RT-MLPA results from eight single cells picked with LCM, bars represent standard deviation. Blood from healthy donor was spiked with 300 cancer cells (SKBR-3). Three single cells (green) had a SKBR-3 like expression profile.

Supplementary Tables

Supplementary Table S1. Summary of MLPA probe, primer and barcode sequences for the genes in the panel

Gene	MLPA probe ID	Chromosomal region	Length (bp)	Left Hybridization Oligonucleotide (LHO)	Spanning Oligonucleotode (SO)	Right Hybridization Oligonucleotide (RHO)	Barcode (5’-3’) attached to 3' end of RHO	Gene specific forward primer	Amount forward primer (pmol per reaction)	Gene specific reverse primer	Amount reverse primer (pmol per reaction)
CD44	S0809-SP0562-L22067	11p13	153	TGCCGCTGAGCCTGGCGCAGATCGATTT	GAATATAACCTGCCGCTTTGCAGGTGTAT	TCCACGTGGAGAAAAATGGTCGCTACAGCATCTC	CATCGCACGAATATAATACA	CTGGGGACTCTGCCTC	1	TCAGCGGCCTCCGTC	1
CDH1	S0793-L07876	16q22.1	129	CCTTGGAGGAATTCTTGCTTTGCTAATTCTGAT	-	TCTGCTGCTCTTGCTGTTTCTTCGGAGGAGAGCG	ATAGGCTGGTTCGTAATCGG	ATTCCTGCCATTCTGGGGATTC	2	CTGGGGGCAGTAAGGGCTCTTT	4
HUWE1	S0797-L22577	Xp11.22	133	CCACCAAGCTGAAGGGCAAAATGCAGAGCAGGTTTGAC	-	ATGGCTGAGAATGTGGTAATTGTGGCATCTCAG	ATTACGACGAACTCAATGAA	TGAGGAGCAGCCACAGA	1	CCACCCAAAGGTCGCT	1
CD24	S0790-SP0560-L22065	06q21	150	CAAGTAACTCCTCCCAGAGTACTTCCAACT	CTGGGTTGGCCCCAAATCCAACTA	ATGCCACCACCAAGGCGGCTGGTGGTGCCCTGCA	TCTACAGGCTCGTATATGTA	CAACAACTGGAACTTCAAGTAA	1	GAAGAGACTGGCTGTTGAC	1
ERBB2	S0794-L08467	17q12	114	CGTTCTGAGGATTGTCAGAGCCTG	-	ACGCGCACTGTCTGTGCCGGTGGCTGTG	CTAAGTAGCCGAATTCCTAG	CCCTGTTCTCCGATGTGTAA	4	GCTCATGGCAGCAGTCAGT	4
KRT19	S0795-SP0469-L20746	17q21.2	170	TGGGCCCTCCCGCGACTACAGCCACTACTACAC	GACCATCCAGGACCTGCGGGACAAGATTCTTGG	TGCCACCATTGAGAACTCCAGGATTGTCCTGCAGATCGACAA	AACCTTAGAGCGGATTAGGG	CTGGTACCAGAAGCAGGG	1	GAAGTCATCTGCAGCCAGAC	1
CDH2	S0808-L21873	18q12.1	165	CAATCCTCCAGAGTTTACTGCCATGACGTT	-	TTATGGTGAAGTTCCTGAGAACAGGGTAGACATCATAGTAGCTAATTTACCGTTGAAATCGATCGATCTAGAT	TTACCGTTGAAATCGATCGA	CATCACAGTGACAGATGTCAATG	4	TGATCCTTATCGGTCACAGTTAG	4

	Universal MLPA primer sequences
**Forward**	Cy3 5’-GGG TTC CCT AAG GGT TGG A-3’
**Reverse**	Bio 5’-GGA CGC GCC AGC AAG ATC CAA TCT AGA-3’

Supplementary Table S2. List of total RNA amounts per cell from SKBR-3 cells

Sample no	Passage	RIN value	Nr of cells	Conc (ng/µl)	RNA amount per cell (pg/cell)
1	10	9	373 125	447	36
2	10	9.6	373 125	367	30
3	10	9.1	373 125	481	39
4	10	9.3	373 125	448	36
5	10	9.1	373 125	427	34

Supplementary Table S3. List of MLPA correction factors

Target gene	Correction factor, combined approach	Correction factor, separate approach
CD24	1,011888972	0,80430375
CD44	2,294580533	1,447755927
HUWE1	0,350093517	0,334803925
CDH1	0,265622474	0,271911396
ERBB2	0,345783858	0,362853984
KRT19	1,647926199	3,93990426
CDH2	0,407163613	0,418107346

Supplementary Table S4. List of FPKM values for the gene panel

Gene	MCF-7	SKBR-3	MDA-MB-231
ERBB2	45,3	865,2	18,7
CDH1	165,9	0,1	0,3
HUWE1	70,3	42,9	60,5
CD24	1321,3	566,9	1,3
CD44	3,0	36,5	346,7
CDH2	0,1	0	7,7
KRT19	1635,6	1686,6	0,6

Supplementary Methods

Detection of gene specific amplicons based on length and barcodes

The array was printed using a Nanoplotter NP-2 (GeSIM, Grosserkmannsdorf, Germany) on activated CodeLink slides (SurModics, Eden Prairie, MN, USA). RT-MLPA products were stained using a primer coupled to Cy3 fluorophore during final amplification. Single-stranded DNA was generated from the double-stranded MLPA products using M-270 Dynabeads coated with streptavidin (Life Technologies, Carlsbad, CA, USA). The 10 µl of magnetic beads were washed with 20 µl 1x Binding & Washing (B&W) buffer (5 mM Tris-HCl, 0.5 mM EDTA, 1 M NaCl), incubated with gentle rotation for 10 minutes in dark in 1x B&W buffer and 5 µl sample (total volume 20 µl). Magnetic beads were washed with 1x SSC buffer (Sigma Aldrich, St Louis, MO, USA) and DNA denatured in 0.1 M NaOH for 10 min in dark. The supernatant containing the forward strands of the MLPA probes were transferred to a new tube, the solution neutralized with 0.1 M HCl, and dissolved in a hybridization buffer with final concentration of 5x SSC buffer (Sigma Aldrich, St Louis, MO, USA) and 0.2% SDS buffer. The single-stranded DNA was loaded onto the array and incubated in the dark for 30 min (50°C) with gentle shaking at 300 rpm. The array was washed with a 50°C solution containing 2x SSC and 0.1% SDS (Sigma Aldrich, St Louis, MO, USA) for 10 min followed by washing with 0.2x SSC for 1 min, 0.1x SSC for 1 min, and finally rinsed in MilliQ water. The arrays were scanned on a DNA microarray scanner with Surescan high-resolution technology (G2565CA) (Agilent, Santa Clara, CA, USA) and the images analysed using GenePix 5.0 software (Molecular Devices, Sunnyvale, CA, USA). The quality of each printed array batch was checked through hybridization of two Cy3 labelled barcode quality oligonucleotides containing sequences complementary to the barcodes for all MLPA products, probe 1 (*ERBB2*, *KRT19*, *CDH2*) and probe 2 (*CD24*, *CD44*, *HUWE1*, *CDH1*). The output signal was normalized against the barcode yielding the strongest signal in that printed array batch. The individual MLPA probe efficiencies in the multiplex reaction were estimated by performing hybridization, ligation and amplification using seven synthetic DNA templates (10 nM) complementary to the hybridizing regions of the MLPA probes. A correction factor was calculated by taking the mean FU for each gene (Y_{1-7}), in four replicates for combined and ten replicates for separate ligation and multiplex amplification, calculating the grand mean (X) from all the gene-specific means (Y_i), then normalizing the values for each individual gene using X and calculating individual MLPA probe efficiencies Z_i ($Z_i = X / Y_i$). The final correction factor $2{*}Z_i$ (2 being a scaling factor) for each gene was multiplied with FU raw data before statistical analysis, see Supplementary Table 3 for list of MLPA correction factors. For patient samples the noise was determined to be the median including three times the standard deviation for the negative controls. The noise was removed from patient samples and negative values were set to zero in the histograms.

RNA library construction, sequencing and data analysis

High-quality RNA (RNA integrity number >9) from the three cell lines was used for sequencing library preparations according to the protocol of the manufacturer (Illumina, San Diego, CA, USA). The libraries were clustered on a cBot cluster-generation system and sequenced as paired-end, 2x100 bp

sequences on an Illumina HiSeq. The sequencing run was performed according to the manufacturer's instructions. The reads were aligned to the human reference genome (hg19) with TopHat[1], the aligned reads were assembled into transcripts using Cufflinks[2] and Fragments Per Kilobase of exon per Million fragments mapped (FPKM) values were calculated. Reads corresponding to gene *CD24* were miss-aligned to *CD24P4* due to gene losses from genome assemblies. Therefore, the expression value for *CD24P4* was used when studying *CD24* expression (NCBI Gene ID 10013394180113). FPKM values for the genes in the gene panel are listed in Supplementary Table 4.

Quantitative PCR
The qPCR was performed using iQ SYBR Green supermix (Bio-Rad, Hercules, CA, USA) for the pre-amplification step using 100 pg of total RNA from MCF-7 cells, and for the final amplification step using single MCF-7 cells picked using LCM. For the latter, separate ligation and amplification was performed where 0.25 µM each of the Y and X primers were added to the iQ SYBR Green Supermix.

Formalin fixation
An in-house formalin fixation method was applied to a few samples. A MNC fraction without MCF-7 cells and a MNC fraction with approximately 1 million MCF-7 cells were spun down and excess liquid was removed. The cell pellets were fixed in 50 µl freshly prepared 2% formaldehyde in PBS (pH 7.4) for 10 min at room temperature. The samples were spun down, the supernatant was removed, and cells were washed with 100 µl PBS. This was repeated twice, before proceeding to IMS. Furthermore, two blood samples (without MCF-7 cells and with approximately 300 000 MCF-7 cells) diluted in PBS were fixed by adding 1 ml of 2% formaldehyde and incubated at room temperature for 10 min before proceeding to IMS.

References

1 Trapnell, C., Pachter, L. & Salzberg, S. L. TopHat: discovering splice junctions with RNA-Seq. *Bioinformatics* **25**, 1105-1111, doi:10.1093/bioinformatics/btp120 (2009).

2 Trapnell, C. *et al.* Transcript assembly and quantification by RNA-Seq reveals unannotated transcripts and isoform switching during cell differentiation. *Nat. Biotechnol.* **28**, 511-515, doi:10.1038/nbt.1621 (2010).
